# A Mass Conserved Reaction–Diffusion System Captures Properties of Cell Polarity

**DOI:** 10.1371/journal.pcbi.0030108

**Published:** 2007-06-08

**Authors:** Mikiya Otsuji, Shuji Ishihara, Carl Co, Kozo Kaibuchi, Atsushi Mochizuki, Shinya Kuroda

**Affiliations:** 1 Department of Anesthesiology, Faculty of Medicine, University of Tokyo, Tokyo, Japan; 2 Division of Theoretical Biology, National Institute for Basic Biology, Okazaki, Japan; 3 Department of Cellular and Molecular Pharmacology and Program in Biological Sciences, University of California San Francisco, San Francisco, California, United States of America; 4 Department of Cell Pharmacology, Graduate School of Medicine, Nagoya University, Nagoya, Japan; 5 Department of Biophysics and Biochemistry, Graduate School of Science, University of Tokyo, Tokyo, Japan; Lawrence Berkeley National Laboratory, United States of America

## Abstract

Cell polarity is a general cellular process that can be seen in various cell types such as migrating neutrophils and *Dictyostelium* cells. The Rho small GTP(guanosine 5′-tri phosphate)ases have been shown to regulate cell polarity; however, its mechanism of emergence has yet to be clarified. We first developed a reaction–diffusion model of the Rho GTPases, which exhibits switch-like reversible response to a gradient of extracellular signals, exclusive accumulation of Cdc42 and Rac, or RhoA at the maximal or minimal intensity of the signal, respectively, and tracking of changes of a signal gradient by the polarized peak. The previous cell polarity models proposed by Subramanian and Narang show similar behaviors to our Rho GTPase model, despite the difference in molecular networks. This led us to compare these models, and we found that these models commonly share instability and a mass conservation of components. Based on these common properties, we developed conceptual models of a mass conserved reaction–diffusion system with diffusion–driven instability. These conceptual models retained similar behaviors of cell polarity in the Rho GTPase model. Using these models, we numerically and analytically found that multiple polarized peaks are unstable, resulting in a single stable peak (uniqueness of axis), and that sensitivity toward changes of a signal gradient is specifically restricted at the polarized peak (localized sensitivity). Although molecular networks may differ from one cell type to another, the behaviors of cell polarity in migrating cells seem similar, suggesting that there should be a fundamental principle. Thus, we propose that a mass conserved reaction–diffusion system with diffusion-driven instability is one of such principles of cell polarity.

## Author Summary

Eukaryotic cells such as neutrophils and *Dictyostelium* cells respond to temporal and spatial gradients of extracellular signals with directional movements. In a migrating cell, specific molecular events take place at the front and back edges. The spatially distinctive molecular accumulation inside cells is known as *cell polarity*. Despite numerous experimental and theoretical studies, its mechanism of emergence has yet to be clarified. We first developed a mathematical model of the Rho small GTP(guanosine 5′-tri phosphate)ases that cooperatively regulate cell polarity, and showed that the model generates specific spatial accumulation of the molecules. Based on our Rho GTPases model and other models, we further established a conceptual model, a mass conserved reaction–diffusion system, and showed that diffusion-driven instability and a mass conservation of molecules that have active and inactive states are sufficient for polarity formation. We numerically and analytically found that molecular accumulations at multiple sites are unstable, resulting in a single stable front–back axis, and that sensitivity toward changes of a signal gradient is specifically restricted at the front of a polarized cell. We propose that a mass conserved reaction–diffusion system is one of the fundamental principles of cell polarity.

## Introduction

Eukaryotic cells such as neutrophils and *Dictyostelium* cells respond to temporal and spatial gradients of extracellular signals with directional movements [1–6]. This process, known as *chemotaxis,* is a fundamental cellular process [5,7–9]. In a migrating cell, specific molecular events take place at the front and back edges [1,2,5,10]. The spatially distinctive molecular accumulation inside cells is known as *cell polarity*. The front–back polarity usually has one axis, and this uniqueness is an important property because a migrating cell with two fronts could not move effectively [11]. Another behavior of the front–back polarity is higher sensitivity of the front to a gradient of extracellular signals [10,12]. This would also be important because the direction of movement should be controlled at the front edge.

Many molecules that are involved in chemotaxis in mammalian cells have been identified [4,5]. Some molecules, including phosphoinositide 3-kinase (PI3K), phosphatidylinositol 3,4,5-triphosphate (PIP_3_), Cdc42, Rac, and F-actin, are specifically localized at the front, whereas others, including phosphatase and tensin homologue deleted on Chromosome 10 (PTEN) and RhoA, are at the back of migrating cells [1,4,10,13–15]. The Rho family of small GTP(guanosine 5′-tri phosphate)ases in particular play a central role in chemotaxis and in establishing cell polarity [15–17]. However, the mechanism of generating spatial accumulation of the Rho GTPases in cell polarity has yet to be clarified.

Many mathematical models that account for gradient sensing and signal amplification in cell polarity have been proposed [12]. The local excitation and global inhibition model has been proposed to explain spatial gradient sensing [6,18]. Some models involve positive feedback loops for amplified accumulation of signaling molecules [19–23]. A reaction–diffusion model that includes local self-enhancement and long-range antagonistic effects has been proposed for directional sensitivity [24]. Most of the reported models of cell polarity, which involve the detailed parameters such as concentrations or rate constants, have been constructed with many parameters and equations. Although these detailed models are at least partially successful in reproducing experimental observations in cell polarity, the theoretical essence underlying cell polarity has not been explicitly demonstrated; thus, a simple conceptual model that can be used for analytical study is needed to extract common principles in cell polarity. Although the reported models consist of distinct molecular species or networks, it should be especially emphasized that many of them are able to exhibit similar behaviors of cell polarity regardless of their different frameworks. This fact indicates that a common principle should underlie the models, and a conceptual model is suitable for extracting common principles in cell polarity.

Because the Rho small GTPases are key regulators for cell polarity [16,17], we first developed a reaction–diffusion model of the Rho GTPases on the basis of an earlier model [25] to examine the spatial properties of the Rho GTPases. We found that the interaction of the Rho GTPases per se can generate specific spatial accumulation of the Rho GTPases, and that our model shows important behaviors of cell polarity. We also found that our model exhibits behaviors similar to the model by Narang and Subramanian [22,23], which is based on the molecular networks that are different from ours. This suggests that common principles should underlie both models. We found that a mass conservation of components and diffusion-driven instability are commonly conserved in the Narang and Subramanian models and in our model. Based on these common properties, we established conceptual models of a mass conserved reaction–diffusion system, and found that such properties can account for the critical behaviors of cell polarity. These results strongly suggest that a mass conservation of components with diffusion-driven instability is one of the fundamental principles of cell polarity.

## Results

### The Rho GTPases Model

We developed a reaction–diffusion model of the Rho GTPases (Rac, Cdc42, RhoA) on the basis of the earlier model of the Rho GTPase [25], which explains the temporal behaviors, to examine whether the interaction of the Rho GTPases can generate the spatial behaviors in the cell polarity of migrating cells. The Rho GTPases exhibit guanine nucleotide–binding activity and function as molecular switches, cycling between an inactive GDP(guanosine 5′-bis phosphate)-bound form and an active GTP-bound form. The Rho GTPases in active forms are located in the plasma membrane, and those in inactive forms are in the cytosol (Figure 1A) [26]. It is likely that molecules in the cytosol have larger diffusivity than those in the plasma membrane. According to some studies, Cdc42 activates Rac [27–29], and RhoA has mutual inhibitory interactions with Cdc42 and Rac [29–33]. In addition, Rac plays a dominant role in a positive feedback loop, which involves PI3K, PIP_3_, and F-actin [13,34–36]. Based on these experimental findings, we developed a diagram of the Rho GTPases interaction (Figure 1B). We assume that molecules of Rac, Cdc42, and RhoA are activated by guanine nucleotide exchange factors (GEFs; *ka_i_*) and are inactivated by GTPase-activating proteins (GAPs; *ki_i_*), and that interactions between molecules (*k_ij_*) are additive to GEFs or GAPs. Some molecule–molecule interactions are stimulation dependent. Activations of molecules by the stimulation (*ks_i_*) are also assumed to be additive to GEFs. As in many previous models [18–23], we describe the spatial kinetics of molecules by simple diffusion equations. A recent study in which the diffusion coefficients of the Rho GTPases in the plasma membrane are determined [37] may support this assumption. The model of the interaction of the Rho GTPases is as follows (see also Materials and Methods):

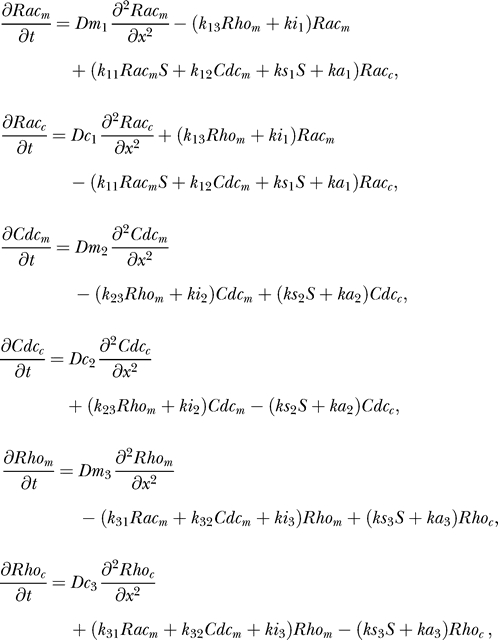
where *Rac*, *Cdc*, and *Rho* with suffixes *m* and *c* denote the concentrations of Rac, Cdc42, and RhoA in the active state (membrane) and inactive state (cytosol), respectively. The numerical suffixes represent the following: 1, Rac; 2, Cdc42; and 3, RhoA. *Dm_i_* and *Dc_i_* denote the diffusion coefficients of molecules in the active state and inactive state, respectively (*Dm_i_* < *Dc_i_*). The position-dependent parameter, *S*, denotes the intensity of stimulation. Because the parameters have not been fully obtained experimentally, we set parameters to reproduce the behaviors of cell polarity (Figure 2), and further analyzed the generality of such behaviors in detail with conceptual models (see below).

**Figure 1:**
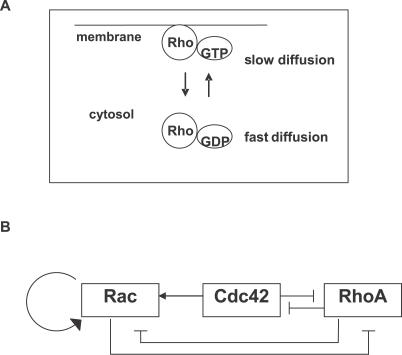
A Reaction–Diffusion Model of the Rho GTPases (A) The Rho family of GTPases, which are localized in the membrane (GTP-bound active forms) or cytosol (GDP-bound inactive forms), has conserved mass and shows slower diffusion in the membrane than in the cytosol. (B) Diagram of the model with the Rho GTPases. Arrows and bars indicate the stimulatory and inhibitory interactions, respectively.

**Figure 2:**
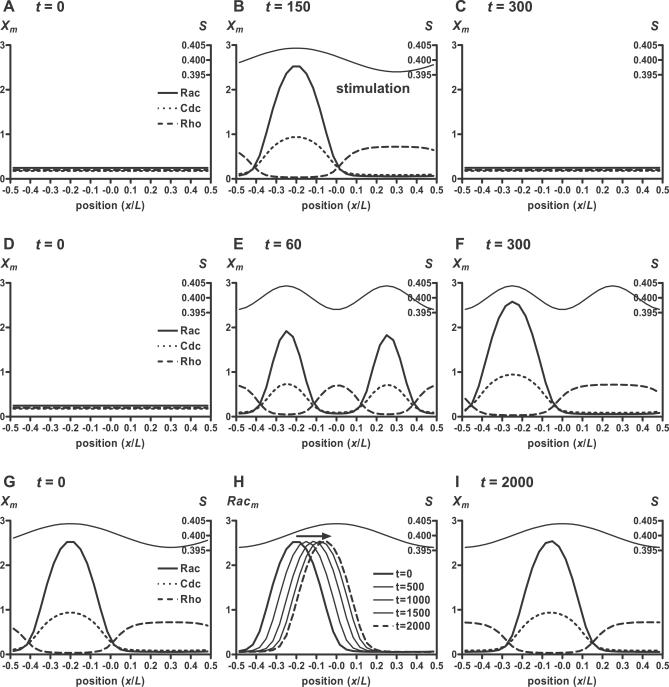
Behaviors of the Rho GTPases Model Spatial profiles of Rac (solid lines), Cdc42 (dotted lines), and RhoA (dashed lines) are shown in (A–G) and (I). The thin lines in (B) and (E–I) indicate the spatial profile of the stimulation. (A–C) Reversible accumulation of the Rho GTPases. We set *S* = 0.1 at *t* = −100, and ran the simulation until *t* = 0 to reach the stationary state (A). Then, the stimulation *S* = 0.4{1 + 0.01cos[2*π*(*x*/L + 0.2)]} was given and the simulation was run until *t* = 150 to reach the stationary state (B). The stimulation point with the maximal intensity was at *x*/*L* = −0.2. The stimulation was reduced to the basal level (*S* = 0.1) again, and the simulation was run until *t* = 300 to reach the stationary state (C). (D–F) Multiple transient, and a single stable, accumulation peaks. Starting with the conditions in (A), we gave random perturbation (±0.01)) to obtain the homogenous initial conditions (D). Then, at *t* = 0, the stimulation *S* = 0.4{1 + 0.01 cos[2*π*(*x*/L + 0.25) × 2]} was given, and the simulation was run for the indicated time (E,F). The stimulation points with the maximal intensity were at *x*/*L* = −0.25 and 0.25. (G–I) Sensing of the stimulation gradient by the polarized peak. We set *S* = 0.4{1 + 0.01 cos[2*π*(*x*/L + 0.2)]} at *t* = −200, and the simulation was run to reach the stationary state where the polarized peak was seen at *x*/*L* = −0.2 (G). The new stimulation *S* = 0.4{1 + 0.01 cos[2*π*(*x*/L)]} was given, and the maximal intensity point was shifted to *x*/*L* = 0. The simulation was run for the indicated time (H,I). The arrow indicates the direction of movement of the polarized peak (H).

#### Reversible accumulation of the Rho GTPases.

Cell polarity accompanies exclusive reversible accumulation of Cdc42 and Rac, or RhoA, at the fronts or backs of migrating cells, respectively [4,10,13–15]. We examined the accumulation of the Rho GTPases in response to a shallow gradient of stimulation (Figure 2A–2C). The basal level of stimulation (*S* = 0.1) did not induce exclusive accumulation of the Rho GTPases (Figure 2A); however, the larger stimulation (averaged *S* = 0.4, with a slight gradient) induced accumulation of Cdc42 and Rac toward the stimulation point with the maximal intensity (*x*/*L* = −0.2), and exclusion of RhoA from the point (Figure 2B). The responses of accumulation and exclusion were switch-like against the intensity of stimulation (unpublished data). The accumulation and exclusion were reversed when the intensity of stimulation reduced to the basal level (*S* = 0.1) (Figure 2C).

#### Multiple transient peaks and a single stable peak.

We next gave two points simultaneous stimulation. Cdc42 and Rac accumulated toward the stimulation points with the maximal intensity (*x*/*L* = −0.25 and 0.25) at *t* = 60 (Figure 2E). However, one of the accumulation peaks eventually disappeared, and only a single peak remained (Figure 2F). This result indicates that the model shows specific behaviors in cell polarity; “uniqueness of axis” (see below). The selection of the remaining peak differed between simulation tests (unpublished data).

#### Sensing of the stimulation gradient by the polarized peak.

We next examined the behavior of the accumulation peak (polarized peak). The polarized peak moved toward the new stimulation point of the maximal intensity, rather than generating a new peak (Figure 2G–2I). This result indicates that the polarized peak can sense a gradient of stimulation (see below).

### Conceptual Model for Cell Polarity

The behaviors in the Rho GTPases model, such as switch-like reversible accumulation, uniqueness of axis, and sensing of the stimulation gradient by the polarized peak, were similar to those observed in the models of Subramanian and Narang [22,23]. Despite the differences of the molecular species and networks among the models, the similarity in behaviors among them raises the possibility that a common principle could underlie them. Therefore, we examined whether common properties can be seen.These models belong to reaction–diffusion systems with a periodic boundary condition and exhibit switch-like response, implying that instability is important for accumulation of the components. In addition, these models involve components whose masses are conserved. The total amount of phosphoinositides between the plasma membrane and the endoplasmic reticulum is conserved in Narang and Subramanian's model [22,23], and the total amounts of the Rho GTPase between the membrane (e.g., *Rho_m_*) and cytosol (e.g., *Rho_c_*) are conserved in our Rho GTPases model. Based on these common properties, we derived a new concept (i.e., mass conserved reaction–diffusion system with diffusion-driven instability) and hypothesized that this system is a fundamental principle of cell polarity. We therefore developed a simple conceptual model with two components (u and v), which belong to a mass conserved reaction–diffusion system with instability, and examined whether the model can cause the behaviors of cell polarity to emerge sufficiently.

#### Mass conserved reaction–diffusion system.

We investigated the following class of reaction–diffusion system, which is composed of two components, u and v:

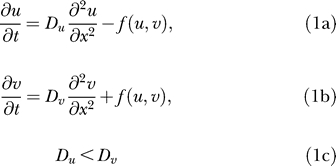
where *u* and *v* denote the concentrations of u and v, respectively, at time *t* and at position *x*, and *D_u_* and *D_v_* denote the diffusion coefficients of u and v, respectively. The reaction term *f* is given by a function of *u* and *v*. Because the total quantity of u and v is conserved in this case, we refer to this system as the *mass conserved reaction–diffusion system*.

We developed several models that belong to the mass conserved reaction–diffusion system and show similar results (unpublished data), and here focused on the following model because of its simplicity for computation.

### Model I



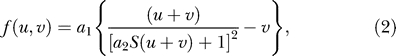
where *a*_1_ and *a*_2_ are parameters of the model. The position-dependent parameter, *S*, is intensity of stimulation (see Materials and Methods). The stability of the homogenous solution in this model depends on the value of *S* (see Materials and Methods).

We found that the conceptual model exhibits behaviors similar to the Rho GTPases model, such as switch-like reversible accumulation (Figure 3A–3C), uniqueness of axis (Figure 3D–3F), and sensing of the stimulation gradient by the polarized peak (Figure 3G–3I). This finding indicates that the conceptual model retains the essential behaviors in the Rho GTPases model. We further used this conceptual model to examine in detail two behaviors of cell polarity: uniqueness of axis and sensitivity of the polarized peak.

**Figure 3:**
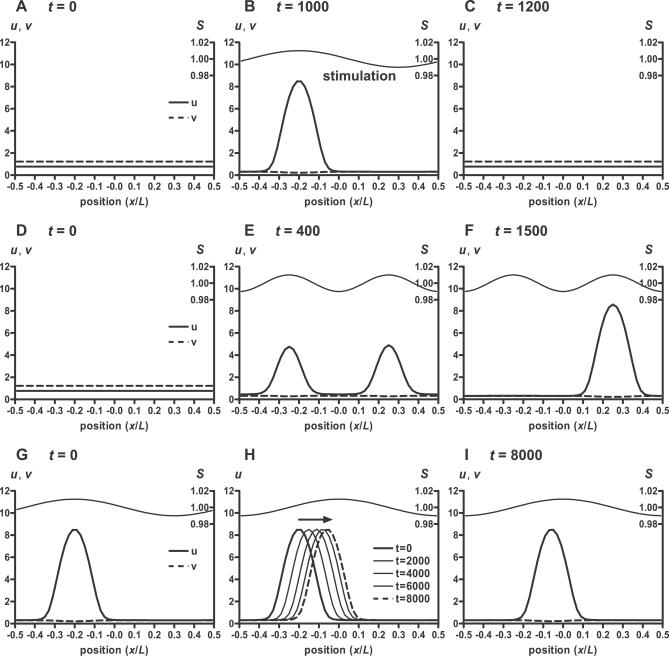
Behaviors of the Conceptual Model I Spatial profiles of *u* (solid lines) and *v* (dashed lines) are shown in (A–G) and (I). The thin line in (B) and (E–I) indicates the spatial profile of the stimulation. (A–C) Reversible accumulation of the components. We set *S* = 0.2 at *t* = −100, and ran the simulation until *t* = 0 to reach the stationary state (A). Then, the stimulation *S* = 1{1 + 0.01 cos[2*π*(*x*/L + 0.2)]} was given, and the simulation was run until *t* = 1,000 to reach the stationary state (B). The stimulation was reduced to the basal level (*S* = 0.2) again, and the simulation was run until *t* = 1,200 to reach the stationary state (C). (D–F) Multiple transient and a single stable accumulation peaks. Starting with the conditions in (A), we gave random perturbation (±0.02) to obtain the homogenous initial conditions (D). Then, at *t* = 0, the stimulation *S* = 1{1 + 0.01 cos[2*π*(*x*/L + 0.25) × 2]} was given, and the simulation was run for the indicated time (E,F). The stimulation points with the maximal intensity were at *x*/*L* = −0.25 and 0.25. (G–I) Sensing of the stimulation gradient by the polarized peak. We set *S* = 1{1 + 0.01 cos[2*π*(*x*/L + 0.2)]} at *t* = −1,000, and the simulation was run to reach the stationary state where the polarized peak was seen at *x*/*L* = −0.2 (G). The new stimulation *S* = 1{1 + 0.01 cos[2*π*(*x*/L)]} was given, and the maximal intensity point was shifted to *x*/*L* = 0. The simulation was run for the indicated time (H,I). The arrow indicates the direction of movement of the polarized peak (H).

#### Numerical evaluation of uniqueness of axis.

We analyzed unstable homogenous solutions to examine the characteristic behaviors of this system by setting large homogenous *S* (*S* = 1) (Figure 4A and 4B). The small perturbation was given to the homogenous stationary state. This perturbation transiently induced two peaks in Model I with *L* = 10. However, one of the peaks eventually disappeared, and only a single peak remained (Figure 4A). When we doubled the system size (*L* = 20), four peaks appeared; however, as in the case with *L* = 10, only a single peak remained and others disappeared (Figure 4B). No peak arose after the first transient peaks appeared. This result suggests that the number of transient peaks depends on the system size, and that multiple transient peaks are unstable, which results in a single peak at the final steady state.

**Figure 4:**
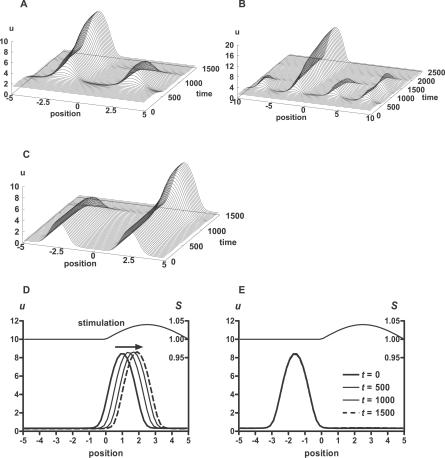
Numerical Evaluation of Uniqueness of Axis and Localization of Sensitivity in Model I (A–C) Numerical evaluation of uniqueness of axis. We set *u* = 1.65, *v* = 0.35, and *S* = 1. These values of (*u*, *v*) were derived from *f*(*u*, *v*) = 0 and *u* + *v* = 2. The system size was taken as *L* = 10 (A) or *L* = 20 (B). We gave random perturbation (±0.01), and the simulation was run for the indicated time (A,B). (C) Next, we obtained a stable single peak in Model I by setting *L* = 5 and taking the initial state as *u* = 1.65 and *v* = 0.35. Because we applied the periodic boundary condition to this system, we could set the center of the concentration peak at *x* = 0 by translation. By duplicating and coupling this profile (*L* = 5), we obtained a new profile (*L* = 10) with two peaks. We gave small perturbation (±0.01) to this profile (*L* = 10), and the simulation was run for the indicated time (C). (D,E) Numerical evaluation of localization of sensitivity. We obtained a stable single peak in Model I by setting *L* = 10 and *S* = 1, and taking the initial state as *u* = 1.65 and *v* = 0.35. We set the center of the concentration peak at *x* = 1.0 (D) or *x* = −1.6 (E) by translation. Then we gave a new stimulation *S* = 1 (*x* < 0) and *S* = 1{1 + 0.04cos[2*π*(*x*/L − 0.25)]} (*x* ≥ 0). The stimulation gradient is given only at the area of *x* > 0. The simulation was run for the indicated time (D,E).

We analyzed the relationship between the number of transient peaks and the system size by linearization analyses around the homogenous states (see Materials and Methods). For Model I (Equation 2), the wave number that grows most rapidly from homogenous state 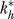 is obtained as 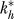 = 1.32; the most growable wavelength is 2π/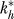 = 4.78. Therefore, the system with *L* = 10 most likely generates two peaks and the system with *L* = 20 four peaks from the homogenous states.

We further confirmed that multiple peaks are unstable. We set a two-peak profile as the initial condition, and examined its stability by giving a small perturbation. As shown in Figure 4C, the two peaks were not stable; one disappeared, and only a single peak remained. This result strongly suggests that multiple peaks are unstable, resulting in a single stable peak.

#### Numerical evaluation of localization of sensitivity.

We examined whether the sensitivity is localized at the polarized peak. We first induced the stable polarized peak as indicated (*t* = 0), and gave the indicated position-dependent stimulation (Figure 4D and 4E). When the polarized peak (peak at *x* = 1.0) overlapped with the gradient of the stimulation, the polarized peak moved along the gradient of *S* (Figure 4D); a new peak did not appear. In contrast, when the polarized peak (peak at *x* = −1.6) did not overlap with the gradient of the stimulation, the polarized peak did not move (Figure 4E); a new peak did not arise. These results indicate that the polarized peak shows sensitivity to a gradient of *S*, whereas the rest of the position does not show such sensitivity, and suggest that the sensitivity is restricted only at the polarized peak.

### Analytical Examination of Unique Axis and Localized Sensitivity

To better understand the results of the numerical simulations, we investigated the following model (see Equations 1a–1c), by analytical approximations:

### Model II



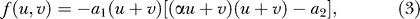
where *D_u_* = *αD_v_*. Here, *a*_1_, *a*_2_, and *α* are parameters of the models. This model belongs to the mass conserved reaction–diffusion system, and is more advantageous for analytical examination. The homogenous solution was unstable regardless of the values of parameters in this model (see Materials and Methods), so this model did not show reversible accumulation. However, the model still retained the important properties such as uniqueness of axis and localization of sensitivity, so we can use this model to analytically examine whether these behaviors can emerge from a mass conserved reaction–diffusion system with instability.

In the following sections, we show that: (1) the model has one-peak stationary states, regardless of the system size (if not too small); (2) multiple-peak stationary states are unstable; and (3) the polarized peak moves depending on the gradient of the parameter value and the sensitivity is localized. Finally, (4), we verified our analyses by comparing analytical results with the values obtained by numerical simulations.

#### Analysis (1): Existence of a one-peak stationary solution.

We define the following variables and function:

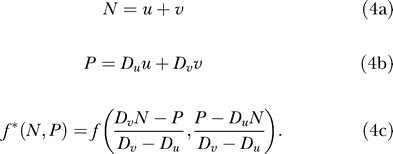

Equations 1a and 1b are rewritten as the following set of partial differential equations for *N* and *P*:

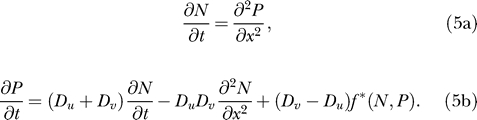
Under the periodic boundary conditions, the stationary solutions of Equations 5a and 5b, *N_e_*(*x*) and *P_e_*(*x*), satisfy the following equations:

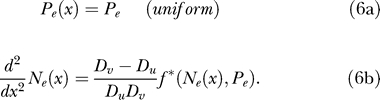
Consider the case of Model II given by Equation 3. For any *P_e_* (>0), Equation 6b with the substitution of Equation 3 has a family of periodic solutions with periods between λ_*min*_ < λ < ∞ (see Materials and Methods). Here λ*_min_* is given by λ*_min_* = 2π{[*D_u_D_v_*/(*D_v_* − *D_u_*)](1/*a*_1_ *a*_2_)}^1/2^. Thus, for an arbitrary large system with *L* > λ*_min_*, one can choose a one-peak solution that satisfies the boundary conditions at *x* = ±*L*/2 by taking λ = *L*. Note that *P_e_* is related to λ and the average mass of *N_e_*(*x*), *N̄* = 1/*L* 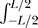 *N_e_*(*x*) *dx*. For a sufficiently long period (λ → ∞), this is expressed as

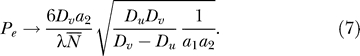
Thus, for a sufficiently large system, *N_e_*(*x*) and *P_e_*(*x*) are approximated as:

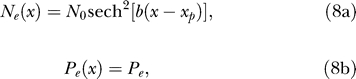
where *x_p_* denotes the center of the peak and *b*, *N*_0_, and *P_e_* are constants given by:

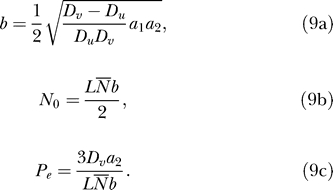
Here we obtain the one-peak solution for Model II (Equation 3) by setting an arbitrary *L* and *N̄*.

#### Analysis (2): Stability of periodic solutions.

We examined the stability of periodic solutions by linearization analysis for a single-peak solution (*Analysis (2.1)*) and multiple-peak solutions (*Analysis (2.2)*), whose periods are equal to the system size and the 1/*n* of the system size, respectively.

*Analysis (2.1): Stability of a one-peak solution.* First, we consider the stability of a one-peak solution given by Equations 8a and 8b. Without loss of generality, we set *x_p_* = 0 here. We set *N*(*x*,*t*) = *N_e_*(*x*) + Δ*N*(*x*,*t*) and *P*(*x*,*t*) = *P_e_* + Δ*P*(*x*,*t*), and the stability is estimated by the linearized equations of Equations 5a and 5b around Equations 8a and 8b, given as follows:

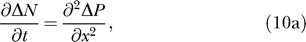


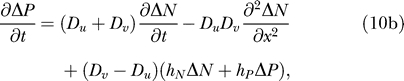
where *h_N_*(*x*) and *h_P_*(*x*) are partial derivatives of f*(*N*, *P*) by *N* and *P*, respectively, at the solution Equations 8a and 8b, that is, *h_N_*(*x*) = ∂f*(*N_e_*(*x*), *P_e_*)/∂*N* and *h_P_*(*x*) = ∂f*(*N_e_*(*x*), *P_e_*)/∂*P*. Let us represent (Δ*N*, Δ*P*) as (*e* ^μ*t*^ *n*_μ_(*x*), *e* ^μ*t*^ *p*_μ_(*x*)) and consider the case of Model II. Equations 10a and 10b lead to the following:

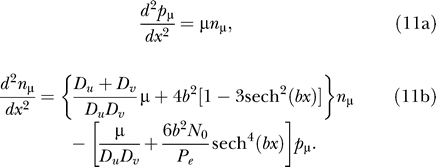
We illustrate *n*_μ_ and *p*_μ_ in Figure 5A and 5B, respectively, for the case with μ > 0 (see Materials and Methods). In the region where |*x*| is larger than 1/*b*, *p*_μ_ can be approximated as follows:

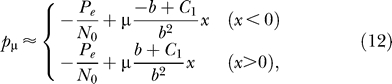
where *C*_1_ is a constant originated from the integral constant. This approximation is verified numerically, as shown by the dashed line with an arrow in Figure 5B. Considering periodic boundary conditions, *p*_μ_(−*L*/2) and *p*_μ_(*L*/2) should connect smoothly; thus, Equation 12 never satisfies Equations 11a and 11b except for μ = 0.

**Figure 5:**
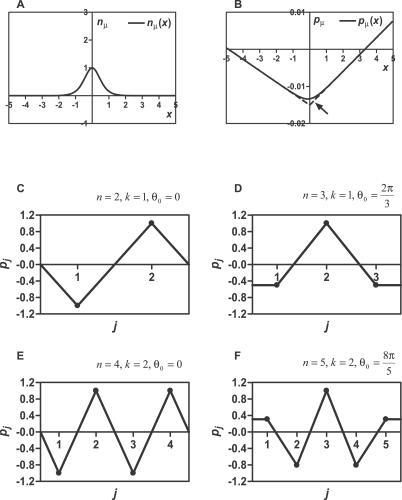
Stability Analysis of Multiple-Peak Solution We seek growable perturbations (*n*_μ_, *p*_μ_) for the periodic solutions as discussed in the text. First, we show the perturbations for the one-peak solution, which are derived from Equations 11a and 11b, without consideration of boundary conditions: (A) *n*_μ_(*x*); and (B) *p*_μ_(*x*). (A,B) We used the following values: *a*_1_ = 0.5, *a*_2_ = 2.2, *D_u_* = 0.1, *D_v_* = 1, *N̄* = 2. Because we cannot determine the values of μ and *C*_1_ without boundary conditions, we arbitrarily set μ = 0.06 and *C*_1_ = 0.3 here. The dashed line with an arrow in (B) indicates the approximation to a piecewise linear function. Next, we show the perturbations for multiple-peak solutions. We can describe a perturbation by a set of *p_j_*, where *p_j_* is the value of p_μ_ at the center of the *j*th peak. As shown in the text, we obtain *p_j_* as 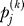 = cos[(2*k*π/*n*)*j* + θ_0_], where *k* is an integer (1 ≤ *k* ≤ *n*/2), corresponding to a mode, and θ_0_ is an arbitrary constant. (C–F) Show the most growable perturbations for two-, three-, four-, and five-peak solutions, respectively. For each *n*, the mode, *k*, that gives the largest value to μ, is determined by μ^(*k*)^ = [(12*n* ^3^ *D_v_a*_2_)/(*L* ^3^ *N̄* ^2^ *b*)]sin^2^(*k*π/*n*).

*Analysis (2.2): Instability of multiple-peak solutions.* Here we consider the case of multiple-peak solutions. As long as *L*/*n* is larger than 1/*b*, an identical *n*-peak periodic solution is approximated by:

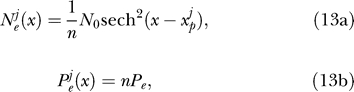
on each *j*th domain defined as 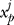 − *L*/2*n* ≤ *x* ≤ 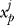 + *L*/2*n*, where 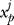 is the position of each peak*_j_* (*j* = 1, 2, ..., *n*) given by 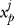 = −*L*/2 + (*j* − 1/2)*L*/*n*. We set Δ*P* = *P*(*x*, *t*) − 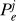 (*x*) = *e* ^μ*t*^ 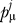 . As in the case of the one-peak solution, we can obtain

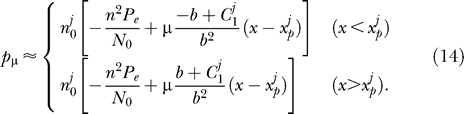
for each *j*th domain. For multiple-peak solutions, 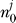 and 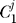 are determined by the boundary conditions such that 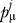 connect smoothly between adjacent domains. This piecewise linear function takes the value *p_j_* = 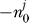 *n* ^2^ *P_e_*/*N*_0_ at *x* = 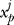 , and at the point the slope changes by −μ(2*N*_0_/*n* ^2^ *P_e_b*)*p_j_*. A smooth connection between them means the slope is given as (*p_j_*_+1_ – *p_j_*)/(*L*/*n*) for 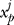 < *x* < 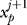 . Thus, *p_j_* satisfies the following relationship:

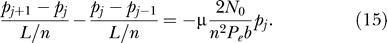
Considering periodic boundary conditions, we obtain the following solutions:

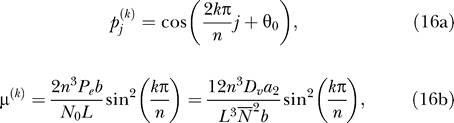
where *k* is an integer (1 ≤ *k* ≤ n/2), corresponding to a mode, and θ_0_ is an arbitrary constant. All modes have positive μ, and therefore can grow. Figure 5C–5F shows the most growable mode for the *n*-peak solution (*n* = 2, 3, 4, 5), that gives the largest value to μ.

Based on this analysis, the one-peak solution is stable, whereas the multiple-peak solutions are unstable, regardless of the parameter values.

#### Analysis (3): Response of the polarized peak to a position-dependent parameter.

Here we study the case in which the system has a position-dependent parameter by substituting 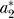 (*x*) = *a*_2_ + ɛ*a*_ɛ_(*x*) for *a*_2_ in Equation 3, where |*εa_ε_*(*x*)| is sufficiently smaller than *a*_2_. It is useful to represent the explicit dependence of f* on the parameter, as f*(*N*, *P*, 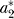 ). In this case, an existing peak moves, as is the case with Model I (Figure 3G–3I). To derive the velocity of the peak (*v_p_*), we define the following variable and functions:

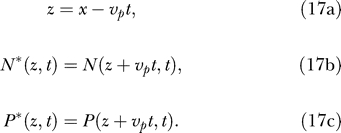

Equations 5a and 5b are rewritten as the following set of partial differential equations for N* and P*:

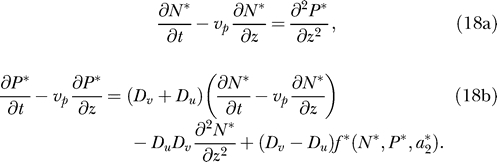
Under the periodic boundary conditions, the stationary solutions of Equations 18a and 18b, 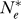 (*z*) and 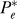 (*z*), satisfy the following equations:

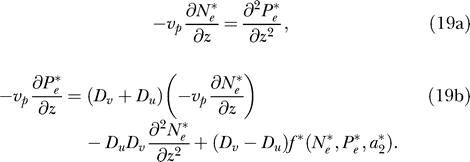
Considering that ε = 0 and *v_p_* = 0 lead to 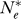 (*z*) = *N*_0_ sech^2^(*bz*) and 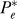 (*z*) = *P_e_*, we can set 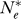 (*z*) = *N*_0_ sech^2^(*bz*) + *n*_ɛ_(*z*) and 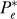 (*z*) = *P_e_* + *p*_ɛ_(*z*) for small value of ε. The linearized approximations of Equations 19a and 19b around (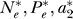 ) = (*N*_0_ sech^2^(*bz*), *P_e_*, *a*_2_) give us the velocity of the peak at *t* = 0 (see Materials and Methods):

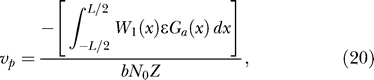
where *W*_1_(*x*) = −2tanh(*bx*)sech^2^(*bx*), and *Z* is a constant. *G_a_*(*x*) is defined by *G_a_*(*x*) = (4*b* ^2^ *N*_0_/*a*_2_)sech^2^(*bx*)*a*_ɛ_(*x*), which reflects the position dependence of 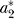 (*x*).

Equation 20 indicates that the velocity of the peak is determined by the integral of *W*_1_(*x*)ɛ*G_a_*(*x*) with respect to *x*. Taking into consideration that ɛ*G_a_*(*x*) represents the position dependence of 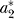 (*x*) and that the position dependence at the site where the value of *W*_1_(*x*)is trivial has little influence on the velocity, we can regard *W*_1_(*x*), which is a kind of weight function, as a “sensing window.” As shown in Figure 6, this window has significant value only at the site of the concentration peak. Indeed, as for the mass conserved reaction–diffusion system, *W*_1_(*x*) is proportional to 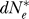 /*dx*, and thus the sensing window is locally open at the preexisting peak, especially at the peripheries of the peak. Note that the integral of *W*_1_(*x*)ɛ*G_a_*(*x*) is zero when *a*_ɛ_(*x*) is an even function; therefore, the sensing window can detect a gradient (or slope) of 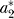 (*x*).

**Figure 6:**
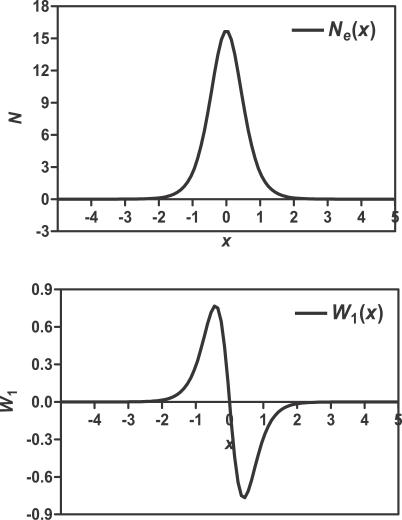
Sensing Window of the Polarized Peak That Detects the Position Dependence of a Parameter The velocity of the polarized peak responding to a parameter gradient is determined by 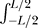 *W*_1_(*x*)ɛ*G_a_*(*x*) *dx*, where ɛ*G_a_*(*x*) represents the position dependence of the parameter. Here we can regard *W*_1_(*x*) as a sensing window. The upper panel shows the profile of the polarized peak, 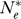 (*x*); the lower panel indicates *W*_1_(*x*).

Based on this analysis, the polarized peak can detect the position dependence of a parameter and move depending on the gradient at the site of the peak. This property can be called “localized sensitivity.”

#### Analysis (4): Verification of analysis by computations.

Finally, in *Analyses (4.1)–(4.3)*, we verified our Analyses (1)–(3), respectively, by comparing analytical results with the values obtained by numerical simulations.

*Analysis (4.1): Approximation of the one-peak solution.* First, we verify the results of Analysis (1): Existence of a one-peak stationary solution. We obtain analytical approximations of the one-peak solution as Equations 8a and 8b, and we compare this approximation with the final profile of the numerical simulation of Model II. The left panel of Figure 7A is the final profile of numerical simulation (see Materials and Methods). The solid line indicates the profile of *N*, and the dashed line indicates *P*. The right panel of Figure 7A shows the approximations given by Equations 8a and 8b, taking the center of the peak as *x_p_* = −2.15. The results of the computations show good agreement with our analytical results. The approximations were also sufficient when we set *L* = 20, 40, and 80 (unpublished data).

**Figure 7:**
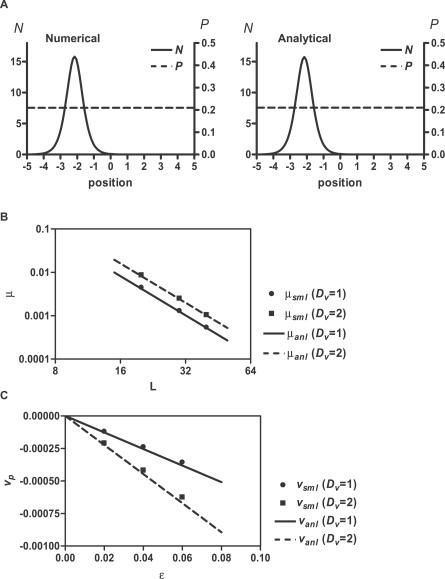
Verification of Analysis by Computations (A) Approximation of one-peak solution. The left panel indicates the result of numerical simulation (Model II); the right panel indicates the analytical approximation. (B) Instability of the two-peak solution. A two-peak state is unstable in Model II and some perturbation grows. We compare the growth rates estimated by analysis with those obtained by simulations. For simulations, we varied *D_v_* (= 1 or 2) and *L* (= 20, 30, or 40); thus, six trials were performed. The axes indicate μ and *L* in double logarithmic scales. (C) Movement of polarized peak is dependent on the parameter gradient. An existent peak moves when a gradient is given to the parameter. We compare the velocity estimated by analysis with those obtained by simulations. For simulations, we varied *D_v_* (= 1 or 2) and ɛ (= 0.02, 0.04, or 0.06); thus, six trials were performed.

*Analysis (4.2): Instability of a two-peak solution.* Next, we verify the results of *Analysis (2.2): Instability of multiple-peak solutions.* According to our analysis, a two-peak solution is unstable, and perturbations will grow exponentially with a growth rate μ*_anl_* given by Equation 16b with *n* = 2 and *k* = 1, that is, μ*_anl_* = (96*D_v_a*_2_)/(*L* ^3^ *N̄* ^2^ *b*). We numerically examined the growth rate of the perturbation given to two-peak solutions with *D_v_* = 1, 2, and *L* = 20, 30, 40 (see Materials and Methods). We logarithmically plotted the growth rates estimated by analytical results (μ*_anl_*; Figure 7B, solid and dashed lines) and those obtained by numerical simulations (μ*_sml_*; Figure 7B, filled circles and squares) against the system size *L*. The results of the analyses and computations were nearly identical.

*Analysis (4.3): Movement of a polarized peak in response to the parameter gradient* **.** Finally, we verify the results of Analysis (3): Response of the polarized peak to a position-dependent parameter.

According to our analysis, the polarized peak will move when a parameter gains position dependence. A concentration peak formed in Model II (Equation 3) with uniform *a*_2_ will move when *a*_2_ is replaced by 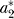 (*x*) = *a*_2_{1 + (ɛ/2)sin[2π(*x*/*L*)]} with a velocity *v_anl_* obtained by Equation 20 as:

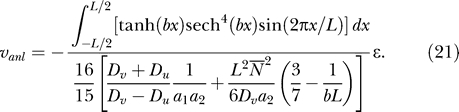
We numerically examined the velocity of movement of the polarized peak in response to a parameter gradient with *D_v_* = 1, 2, and ɛ = 0.02, 0.04, 0.06 (see Materials and Methods). We plotted the velocities estimated by analytical results (*v_anl_*; Figure 7C, solid and dashed lines) and those obtained by numerical simulations (*v_sml_*; Figure 7C, filled circles and squares) against the parameter gradients ɛ. The results of the analyses and computations were nearly identical.

## Discussion

### Rho GTPases and Conceptual Model

In this study, we used a mathematical model to clarify the role of the interaction between the Rho GTPases in cell polarity and developed a conceptual model of cell polarity to glean a theoretical understanding of the unique behaviors of cell polarity.

The Rho GTPases regulate the remodeling of the actin cytoskeleton via actin polymerization, depolymerization, and myosin activity [15,17,26], which ultimately establishes cell polarity. Then, what regulates the spatial activity of the Rho GTPases? The model proposed by Sakumura et al. indicates that the interaction of the Rho GTPases can regulate their own temporal activities [25]. We demonstrated that the interaction of the Rho GTPases can regulate their own spatial activities. In reality, the interaction of the Rho GTPases can provide more complicated temporal and spatial regulation of their activities. Further study, including the determination of kinetic parameters of the interaction, is necessary to develop a more realistic model of the Rho GTPases.

The activator–inhibitor model for pattern formation [38] and the local excitation and global inhibition model for directional sensing in chemotaxis [6] belong to conceptual models, rather than to detailed biological models. Such conceptual models with reduction of equations and parameters make analysis simpler and clearer. We identified a mass conserved reaction–diffusion system with instability as common properties between the cell polarity models. The model belonging to this system can sufficiently reproduce the important behaviors of cell polarity, such as uniqueness of axis and localization of sensitivity, and enabled us to theoretically understand such behaviors, which are difficult to examine without the models.

### Uniqueness of Axis and Localization of Sensitivity

When interleukin-8, a chemoattractant, is applied simultaneously from two directions at a 45° angle, normal neutrophils choose one direction for migration instead of responding to both sources [11]. Neutrophils with multiple leading edges are rarely observed under normal conditions [39]. When HL60 cells are transfected with a dominant-negative Rho construct or treated with Rho-kinase inhibitors, many cells exhibit the multiple pseudopods, where, in some cells, protrusions gradually withdraw, leaving a single, prominent pseudopod [10]. In addition, inhibitions of PI3Ks cause HL-60 cells to form multiple pseudopods, which are weak and transient [39]. These results suggest the instability of multiple leading edges, which may make the front of a migrating cell single and stable. Chemotactic cells must have only one front–back axis because multiple fronts would prevent fine migration. Subramanian and Narang investigated the response of their model to two almost identical stimulations [23] and showed that only one of the two peaks that arise persists, which agrees with our results. Here we show that uniqueness of axis emerges from instability of multiple peak solutions in a mass conserved reaction–diffusion system (Figures 4C and 5).

In neutrophils [40], HL-60 cells [10], and *Dictyostelium* cells [41], the polarized cells respond to changes in direction of a gradient by performing U-turns rather than by simply reversing polarity. In addition, polarized migrating cells move forward without responding to the chemoattractant source near their rears [12]. These experimental findings indicate that the sensitivity to chemoattractants is localized at the leading edge of polarized cells. The localized sensitivity focuses the activity of the actin cytoskeleton at the leading edge, resulting in faster movement toward a chemoattractant source [12]. Few mathematical theories, however, have been proposed to explain the localization of sensitivity. Here we show that localization of sensitivity depends on the specific localization of a sensing window at the polarized peak in a mass conserved reaction–diffusion system (Figure 4D and 4E and Figure 6). It should be added that many other systems can also exhibit a localized sensing window.

### Biological Interpretation of *Mass Conservation*

Consider a molecule that satisfies the following conditions: (1) the molecule (X) has two states (Xm and Xc); (2) the total amount of X is conserved; and (3) the diffusion coefficient of Xc is larger than that of Xm. Two states of this molecule can be treated as components of a mass conserved system. Some kinds of small GTPases, such as those of the Rho family, have two forms, active and inactive forms; the Rho GTPases in the active forms are located in the membrane, and those in the inactive forms are in the cytosol [26]. Some enzymes involved in the cell polarity of chemotactic cells, such as PI3K and PTEN, are also reported to show a relationship between their activity and membrane binding [42–46]. Molecules in the cytosol may well diffuse faster than those in the plasma membrane. Thus, these molecules can be considered as components of mass conserved systems.

Chemotactic cells, such as *Dictyostelium* cells and neutrophils, polarize within a few minutes (30 s to 3 min) after they are exposed to chemoattractants [6,10,47]. Because the time scale of cell polarity is likely to be much shorter than that of gene expression and protein synthesis, we can assume that the masses of molecules are constant during the polarization of chemotaxis.

### Instability of Multiple-Peak Solutions

We numerically and analytically show that multiple-peak solutions are unstable. To facilitate an understanding of the physics of this instability, we attempt to give an intuitive physical explanation of the behavior of molecules in the case where there exist two peaks (Figure 8), just as in Figure 4C. We simplify the situation as follows. (1) There are two spaces (each space has one peak). (2) The molecules have two forms, u and v, which have small and large diffusivities, respectively. (3) No molecule is generated or degraded in the spaces. (4) The molecules move between spaces, mainly in v-form, depending on the concentration gradient of v-form molecules. (5) The u-form molecules convert v-form molecules into u-form, and this positive feedback is so strong that infusion of molecules into the space causes a decrease in v-form molecules. Here, consider that a few molecules move from one space (*S*1) to the other (*S*2). According to (5), because of the nature of the positive feedback, the number of v-form molecules in *S*2 declines as the total number of molecules in *S*2 increases. In turn, according to (4), the declining of the number of v-form molecules in *S*2 successively facilitates further transfer of v-form molecules from *S*1 to *S*2, resulting in the further increase of the total number of molecules in *S*2. This flux is never disrupted by the generation or degradation of molecules because of the mass conservation. Therefore, two peaks in such a system are unstable. The condition (5) seems critical for instability, and we analyzed such conditions mathematically elsewhere [48].

**Figure 8:**
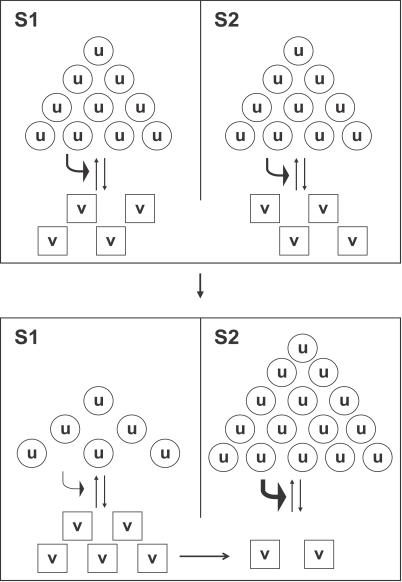
Schematic of Unstable Multiple-Peak Solution in a Mass Conserved Reaction–Diffusion System We set the two-peak solution (*S*1 and *S*2, top panel), which is similar to Figure 4C. We consider the following conditions. There are two spaces (each space has one peak). The molecules have two forms, u and v, which have small and large diffusivities, respectively. No molecule is generated or degraded in the spaces. The molecules move between spaces, mainly in v-form, depending on the concentration gradient of v-form molecules. The u-form molecules convert v-form molecules into u-form, and this positive feedback is so strong under the condition where ∂*P_e_*/∂* N̄* < 0 [48] that infusion of molecules into the space causes a decrease in v-form molecules. Infusion of molecules to *S*2 accompanies the removal of the same number of molecules from *S*1 because of the mass conservation (bottom panel).

### Specific Feature of the Mass Conserved Reaction–Diffusion System

One of the most extensively studied reaction–diffusion models is the Turing model, in which robust spatial patterns, such as stripes or spots, emerge via a diffusion-driven instability [38,49]. An ordinary Turing pattern in 1-D space is stripes with an intrinsic scale length [50]. Mass conserved models also generate multiple peaks from the homogenous state during the early phase, which is explained by diffusion-driven instability. However, they exhibit characteristic transitions after the initial peaks arise: most peaks become smaller and eventually disappear, and only one peak persists (Figure 4A and 4B). Why is the behavior of the mass conserved system so different from that of ordinary Turing models? Consider a reaction–diffusion system with vast size (*L* → ∞) and interval *I* [*x*_1_, *x*_2_] within the system, where *x*_1_ and *x*_2_ are arbitrary but far apart. Can we predict what will happen to interval *I*? For an ordinary Turing model, the linearization analysis around the homogenous solution gives us sufficient information [50]. The mass conserved system is more complex, however, because the behavior differs between the case where the components flow into interval *I* versus the case where they flow out, and we cannot predict which will occur. This difference in predictability seems to be fundamentally linked to the different behavior and the specificity of the mass conserved system. Mass conserved models have multiple stationary states that are spatially homogenous or periodic, including a one-peak state and multiple-peak states. We show that the multiple-peak stationary states are unstable, resulting in a single stable peak.

In some reaction–diffusion systems, such as the activator–inhibitor model (or substrate–depleted model), high diffusivity of inhibitor (or substrate) make multiple peaks unstable, resulting in a single stable peak [51]. An intuitive explanation for this instability is that an inhibitor, which is generated at the peak, rapidly diffuses throughout the cell. In the cell where the inhibitor can be generated and degraded, the spread of the inhibitor requires the large diffusivity to overwhelm the inhibitor degradation. On the other hand, in a mass conserved reaction–diffusion system, where no molecule is generated or degraded, a peak takes up molecules from its surroundings to grow. That is, the growing peak inhibits the system not by spread of inhibitor but by deprivation of molecules. In this case, large diffusivity is not required because there is no competitor to overcome, such as generation of molecules, and the inhibition can eventually spread throughout the cell. Indeed, Equation 16b clearly indicates that any *D_v_* can make multiple peaks unstable (μ > 0), at least in Model II.

It may be counterintuitive that any mass conserved system finally exhibits a one-peak pattern. For Model II, the final steady state was a one-peak solution regardless of the system size, even when *L* was infinitely large. But for Model I, the final steady state had two peaks when we set *L* = 80 (unpublished data). Some mass conserved models probably have a maximum size to have a unique peak. However, this maximum size is independent of the linearization analysis. The conditions for the uniqueness of concentration peak will be elucidated in future analyses.

### Future Perspective and Conclusion

Because properties observed in simple models are expected to be conserved in more detailed models, we assume that movement of molecules follows a simple diffusion equation in our conceptual model. Active transport systems, such as actomyosin- and microtubule-based active transports, regulate cell polarity in various cellular processes [52]. Such active transports are likely to follow the formation of intracellular asymmetry, which takes place under the resting condition where the cell polarity is yet to be generated. Under such conditions, the diffusion of the Rho small GTPases has been measured and shown to be approximated by an apparent simple diffusion, if viewed on the order of seconds or tens of seconds [37]. Because our concern in this study is the earlier asymmetry formation rather than the completion of the polarity, which includes active transport, we here assumed the simple diffusion of the Rho GTPases. However, we will readily incorporate the detailed mechanism of the transport system of the Rho–GTPases in a future model.

In this study, we focused on the stationary state, but not on the transient state, which involves adaptation in response to a transient signal [44,53–55]. Such properties, as well as high dimensionality and multiple components, should be incorporated into a future model.

Although our model is rather simple, it shows the important properties of cell polarity such as switch-like reversible response, uniqueness of axis, and localization of sensitivity. We further demonstrated that the instability of multiple-peak solutions and the specific localization of a sensing window at the polarized peak in a mass conserved reaction–diffusion system are responsible for uniqueness of axis and localization of sensitivity, respectively. One remarkable feature of a mass conserved reaction–diffusion system compared with other models is that a mass conserved reaction–diffusion system does not require strict assumptions for diffusion coefficients, such as smaller and much larger diffusivities of excitatory and inhibitory molecules, respectively, in the local excitation and global inhibition models [6,18], or an extraordinary large diffusivity of the inhibitor in the Gierer–Meinhardt model [51]. Since the Rho GTPases, PI3K, or PTEN have thus far not been demonstrated to involve such ad hoc assumptions of diffusivity, a mass conserved reaction–diffusion system is more likely to explain cell polarity where these molecules are involved, and to be adapted to a wide range of cell polarity. Taking into consideration that the Rho GTPases system satisfies conditions of a mass conserved reaction–diffusion system, it is likely that this system is one of the fundamental principles of cell polarity.

## Materials and Methods

### Simulation.

We considered a one-dimensional circular system with circumference *L*. The position is represented by *x* (−*L*/2 ≤ *x* ≤ *L*/2). We applied the periodic boundary condition, which is used in some models that explain cell polarity [23,24]. We used explicit difference methods to perform simulations. The difference intervals for calculations are shown in the following text.

### Simulation of the Rho GTPases model.

The parameter values were set as follows: *L* = 10, *Dm_i_* = 0.04, *Dc_i_* = 3 (*i* = 1, 2, 3), *ks*_1_= 1, *ks*_2_ = 1, *ks*_3_ = 1, *ka*_1_ = 0.2, *ka*_2_ = 0.2, *ka*_3_ = 0.2, *ki*_1_ = 0.4, *ki*_2_ = 0.2, *ki*_3_ = 0.2, *k*_11_ = 4, *k*_12_ = 3, *k*_13_ = 5, *k*_23_ = 6, *k*_31_ = 4, and *k*_32_ = 2. The difference intervals for calculations were taken to be Δ*t* = 0.01 and Δ*x* = 0.33. We set *X_m_*(*x*) = 0.3, *X_c_*(*x*) = 0.7 (*X* = *Rac*, *Cdc*, *Rho*), unless specified.

### Simulation of Model I.

The parameter values were set as follows: *a*_1_ = 2.5, *a*_2_ = 0.7, *D_u_* = 0.01, *D_v_* = 1, and *L* = 10. The difference intervals for calculations were taken to be Δ*t* = 0.01 and Δ*x* = 0.2. We set *u* = 1 and *v* = 1, unless specified.

### Simulation of Model II for Analysis (4).

The parameter values were set as follows: *a*_1_ = 0.5, *a*_2_ = 2.2, *D_u_* = 0.1, *D_v_* = 1 *or* 2, *N̄* = 2. The difference intervals for calculations were taken to be Δ*t* = 0.005 and Δ*x* = 0.2.

*Approximation of a one-peak solution.* The computation was performed by setting *L* = 10 and *D_v_* = 1 and taking the initial state as *u* = 1 and *v* = 1 with small perturbation (±0.01). The final profile (*t* = 200) is shown in the left panel of Figure 7A. The solid line indicates the profile of *N*, and the dashed line indicates *P*.

*Instability of a two-peak solution*. We examined the instability of two-peak solutions. First, we obtained a stable one-peak pattern in Model II (Equation 3) by taking the size to be *L*/2, where *L* = 20, 30, 40, and taking the initial state as *u* = 1 and *v* = 1 with small perturbation (±0.01). Because we applied the periodic boundary condition to this system, we could set the center of the concentration peak at *x* = 0 by translation. Next, by duplicating and coupling this profile (*L*/2), we obtained a new profile (*L*) with two peaks. We used this profile (*L*) with small perturbations (±0.01) as the initial state of the following simulation. All trials (*D_v_* = 1, 2 and *L* = 20, 30, 40) showed instabilities of two-peak profiles, and we obtained the growth rate, μ*_sml_*, from the change in peak height.

*Movement of a polarized peak in response to the parameter gradient*. We examined the response to the parameter gradient. First, we obtained a stable one-peak pattern in Model II (Equation 3) by setting *L* = 10 and taking the initial state as *u* =1 and *v* = 1. We set the center of the concentration peak at *x* = 0 by translation. Next, we substituted 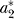 (*x*) = *a*_2_{1 + (ɛ/2)sin[2π(*x*/*L*)]} for *a*_2_ in Equation 3. All trials (*D_v_* = 1, 2 and ɛ = 0.02, 0.04, 0.06) showed movement of the polarized peaks, and we obtained the velocities, *v_sml_*, from the results.

### Linearization analysis around the homogenous state.

In the homogenous stationary state, the Jacobian matrix for the reaction terms is given by

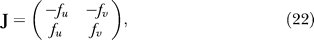
where *f_u_* and *f_v_* denotes the partial derivatives of *f* by *u* and *v*, respectively, at a homogenous stationary state of Equations 1a and Equations 1b. We obtained a condition for instability of the homogenous solution: (*D_u_f_v_* − *D_v_f_u_*)/(*D_u_D_v_*) > 0. For example, the homogenous solution is stable with *S* = 0.2 in Model I, whereas unstable with *S* = 1, under the following conditions: *a*_1_ = 2.5, *a*_2_ = 0.7, *D_u_* = 0.01, *D_v_* = 1, *u* + *v* = 2. The homogenous solution in Model II is always unstable.

Through the stability analysis using J, the range of wave numbers (*k_h_*) that have positive eigenvalues is obtained as 0 < *k_h_* < [(*D_u_f_v_* − *D_v_f_u_*)/(*D_u_D_v_*)]^1/2^, and the wave number that has the largest eigenvalue and grows most rapidly from the homogenous state, 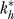 , is obtained as follows:

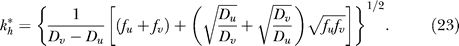
For Model I (Equation 2), we obtain 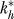 = 1.32 under the following conditions: *a*_1_ = 2.5, *a*_2_ = 0.7, *D_u_* = 0.01, *D_v_* = 1, *S* = 1, *u* + *v* = 2.

### Periodic solution of Equation 6b.

Equation 6b has the same formulation as classical Newton mechanics. We define *V*(*N_e_*) as

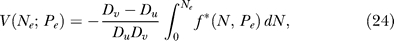
and Equation 6b implies

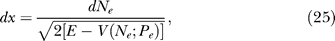
where E is a constant value, corresponding to period and total mass of *N_e_*(*x*). The period λ and the average mass *N̄* = (1/λ) 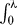 *N_e_*(*x*)*dx* satisfy the following equations:

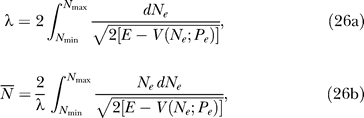
where *N_min_* and *N_max_* are minimum and maximum levels of *N_e_*(*x*), respectively, and are derived from *V*(*N_min_*) = *V*(*N_max_*) = *E* (0 < *N_min_* < *N̄* < *N_max_*). Equation 26a and Equation 26b give the relationship among *P_e_*, λ, and *N̄*, where *N̄* is straightforwardly derived from the initial condition of (*u*, *v*). For Model II (Equation 3),

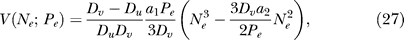
and *E* can range between E_*_ < *E* < 0 for *N_e_*(*x*) to be a periodic solution. Here E_*_ = −{[(*D_v_* − *D_u_*)/*D_u_D_v_*][(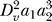 )/6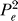 ]}. As *E* becomes smaller (*E* → E_*_), the period λ converges to λ*_min_*, which is the shortest wavelength in the periodic solutions. As *E* becomes larger (*E* → 0), the period λ diverges. The solution of *N_e_*(*x*) at *E* = 0 corresponds to the separatrix of Equation 6b, indicating an infinite period (λ → ∞). The explicit form of *N_e_*(*x*) for *E* = 0 can be obtained by

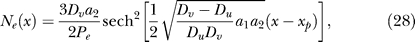
which has the sole peak at *x* = *x_p_* and decays to zero as *x* → ±∞. For a sufficiently large system, Equation 28 is a good approximation of the solution for −*L*/2 < *x* < *L*/2. Equations 28 and 7 lead to Equations 8a and 8b, and 9a–9c.

### Solution of Equations 11a and 11b.

If there is nontrivial (*n*_μ_(*x*), *p*_μ_(*x*)) that satisfies Equations 11a and 11b for μ with a positive real part, the solution is unstable. Note that (*n*_μ0_, *p*_μ0_) = (*n*_0_sech^2^(*bx*), − *n*_0_ *P_e_*/*N*_0_) satisfies Equations 11a and 11b for μ = 0 under periodic boundary conditions. Here *n*_0_ is an arbitrary factor, originated from the linearity of equations, and we can set *n*_0_ = 1. For μ with an absolute value near zero, we can obtain (*n*_μ_, *p*_μ_) by the expansion from (*n*_μ0_, *p*_μ0_) with regard to μ. To do this, we take *n*_μ_ = *n*_μ0_ + μ*n*_μ1_ + … and *p*_μ_ = *p*_μ0_ + μ*p*_μ1_ + … . In the first order of the expansion, (*n*_μ1_, *p*_μ1_) obeys the following equations:

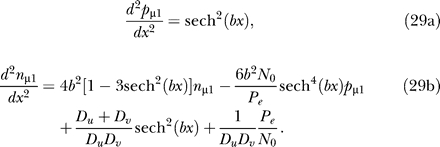
Thus, *p*_μ_ is immediately derived from Equation 29a as:

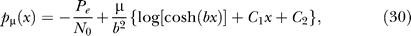
where *C*_1_ and *C*_2_ are integral constants. We can obtain *n*_μ_ by solving by solving Equation 29b with substitution of *p*_μ1_. *C*_2_ is determined by the mathematical condition that *n*_μ1_ should be orthogonal to *n*_μ0_. In practice, *C*_2_ has little influence on (*n*_μ_, *p_u_*), and we set *C*_2_ = 0 in the analysis.

### Derivation of *v_p_* from Equations 19a and 19b.

The linearized approximations of Equations 19a and Equations 19b are given as follows:

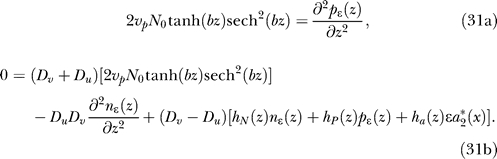
where *h_N_*(*z*) = ∂f*(*N_e_*(*z*), *P_e_*,*a*_2_)/∂*N*, *h_P_*(*z*) = ∂f*(*N_e_*(*z*), *P_e_*,*a*_2_)/∂*P*, *h_a_*(*z*) = ∂f*(*N_e_*(*z*), *P_e_*,*a*_2_)/∂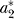 . Because *t* is no longer a variable, we set *t* = 0 without loss of generality and replace *z* with *x* in the following analysis. Equation 31a under the periodic boundary condition leads to *p*_ε_ as follows:

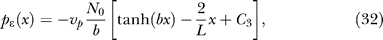
where *C*_3_ is an integral constant. By substituting, Equation 32 into Equation 31b, we obtain the following:

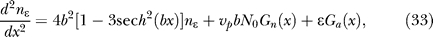
where *G_n_*(*x*) and *G_a_*(*x*) are defined by:

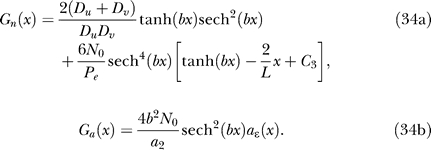
By solving Equation 33, we obtain *n*_ɛ_:

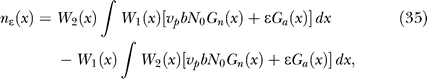
where *W*_1_(*x*) and *W*_2_(*x*) are defined by

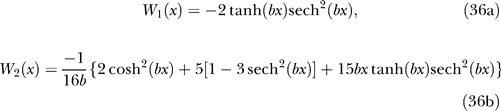
Considering the periodic boundary condition for *n*_ɛ_(*x*), we obtain the following equation for sufficiently large *L* (solvable condition):

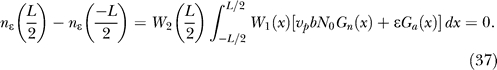
This leads to the velocity of the peak:

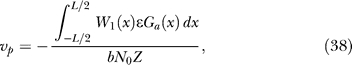
where *Z* is given as follows:

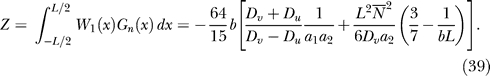



## Abbreviations

GAP: GTPase-activating proteins
GDP: guanosine 5′-bis phosphate
GEF: guanine nucleotide exchange factor
GTP: guanosine 5′-tri phosphate
PI3K: phosphoinositide 3-kinase
PIP_3_: phosphatidylinositol 3,4,5-triphosphate
PTEN: phosphatase and tensin homologue deleted on Chromosome 10
u and v: components of the model
*u* and *v*: concentrations of components u and v
